# Functional diversity in bacterial communities of an integrated constructed wetland used for *in situ* bioremediation of sewage

**DOI:** 10.3389/fmicb.2026.1803785

**Published:** 2026-05-28

**Authors:** Nidhi Seth, Mansi Bansal, Saurav Mazumdar, Sudeshna Mazumdar-Leighton, Suman Lakhanpaul, Sharad Vats, Yasir Arafat, C. R. Babu

**Affiliations:** 1 Centre for Environmental Management of Degraded Ecosystem, University of Delhi, Delhi, India; 2 Department of Botany, University of Delhi, Delhi, India; 3 TapAI LLC, Washington, DC, USA; 4 Department of Bioscience and Biotechnology, Banasthali Vidyapith, Rajasthan, India

**Keywords:** constructed wetland, functional diversity, microbial community, pollutant removal, sewage, stabilization pond

## Abstract

Constructed wetlands (CWs) offer effective, economical, environment-friendly and energy-efficient solution to growing challenges of increasing sewage and wastewater loads in urban areas. Although microbial communities form an integral component of constructed wetlands for sewage treatment, functional processes and their dynamics during sewage bioremediation in constructed wetlands remain largely uncharacterized. Moreover, the association of specific bacterial taxa with remediation of different sewage and water quality parameters remains largely unclear. This study explored the functional diversity likely associated with microbial communities of a constructed wetland system used for *in situ* remediation of 1 MLD (Million Liters per Day) sewage without external energy input since 2014. Different bacterial functional groups in the sludge from a stabilization pond and from rhizospheric sediments of the integrated constructed wetland were predicted using a 16S rRNA gene metagenomic sequencing dataset. Correlation analysis, multivariate statistics and a co-occurrence network were used to assess the bacterial groups associated with changes in water quality as it flows through different components of the integrated CW and highlight association patterns predicting major exchanges which might be operating in the microbial communities. While stabilization pond microbiome was dominated by bacterial groups such as *Firmicutes, Desulfobacterota* and *Methylomirabilota* known to be involved in carbon fermentation, sulphate reduction and methanogenesis, the rhizospheric sediments showed prevalence of bacteria associated with nitrogen reduction including *Nitrospirota* and *Planctomycetota* contributing to improved sewage quality parameters. Such results indicated complex microbial interactions involving bacteria from diverse functional groups sustaining bioremediation in the CW. The identification of primary bacterial taxa along with their putative functions can help in designing strategies to improve sustainable, nature-based wastewater treatment by CW systems.

## Graphical Abstract

**Figure:**
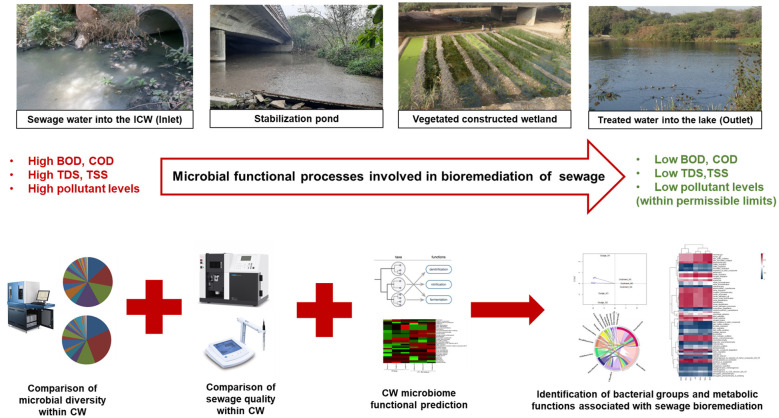

## Introduction

Increasing loads of wastewater (including sewage) emphasize the necessity of sustainable and reliable alternatives to conventional sewage treatment plants (STPs) for bioremediation of sewage in dense urban hubs. Constructed wetlands can contribute significantly to recycle and reuse sewage wastewater with zero or negligible energy input (Rajan et al., 2018; Vymazal et al., 2021). Furthermore, CWs do not generate hazardous residues that can further add to the perils of waste management and are easy to operate and maintain (Vymazal et al., 2021; Kushwaha et al., 2024). CWs can facilitate in achieving the sustainable development goal of United Nations Sustainable Development Goals (2020)—reducing the discharge of untreated water into water bodies to half by 2030 (United Nations Sustainable Development Goals, 2020). Understanding the diversity of functional groups in microbial communities can help identify bacterial taxa for optimizing the functioning of CWs and development of suitable microbial consortia that enhance pollutant removal efficiency (Gaikwad et al., 2014; Adamu et al., 2023; Shan et al., 2023; Kushwaha et al., 2024).

CWs mainly involve physical, chemical and biological processes resulting from interactions among substrate, aquatic plants, and microorganisms to achieve the bioremediation of wastewater. In 1950s, the first experimental study on wastewater treatment by CW using aquatic macrophytes was carried out in Germany (Vymazal, 2005). Two decades later, the ecological theory of CW based on “Root-Zone-Method” was eventually proposed (Kickuth, 1977). Using these principles, several kinds of constructed wetlands have been designed till date with variations in hydrological conditions, kinds of plants and vegetation types and directions of water flow for treatment of wastewaters. Although different kinds of constructed wetland systems have been developed according to specific needs and site conditions, all CWs are a composite, designed ecosystems consisting of substrate, media, water, aquatic plants and microbes (Rajan et al., 2018; Vymazal et al., 2021; Kushwaha et al., 2024; Ali et al., 2024).

Microbial communities in constructed wetlands are crucial for the removal of organic and inorganic pollutants like nitrogen (ammonium, NH_3_-N; nitrate, ${\text{NO}}_{3}^{-}$; nitrite, ${\text{NO}}_{2}^{-}$), phosphorus (phosphate, PO_4_-P), harmful contaminants from the untreated waste water including pharmaceuticals, chemical pesticides, heavy metals and pathogenic bacteria (Kurzbaum et al., 2012; Wang et al., 2022; Ali et al., 2024). High-throughput sequencing methods have allowed comprehensive profiling of microorganisms in constructed wetlands including both culturable and unculturable forms. The 16S rRNA gene metagenomic sequencing data can also be used to predict functional information for microbial communities to get an insight on the underlying biogeochemical processes that allow bioremediation of sewage wastewater in CW and enable availability of clean water. Correlation analyses between co-occurring microbial taxa and different environmental factors can further help elucidate how microbial communities respond to changes in physicochemical parameters of sewer water and thus help in the optimization of wetland performance and pollutant removal efficiency (Adamu et al., 2023; Shan et al., 2023; Ali et al., 2024). The precise detection of microorganisms that are indicators of pollution and fecal contamination in constructed wetlands can also help in honing strategies for monitoring and managing pollutant loads in CWs (Gaikwad et al., 2014; Alabiso et al., 2023; Adamu et al., 2023; Shan et al., 2023; Kushwaha et al., 2024). Such findings can also lead to identification of taxa which can serve as putative candidates for development of microbial consortia to enhance the performance of CWs (Adamu et al., 2023; Shan et al., 2023).

**Figure 1:**
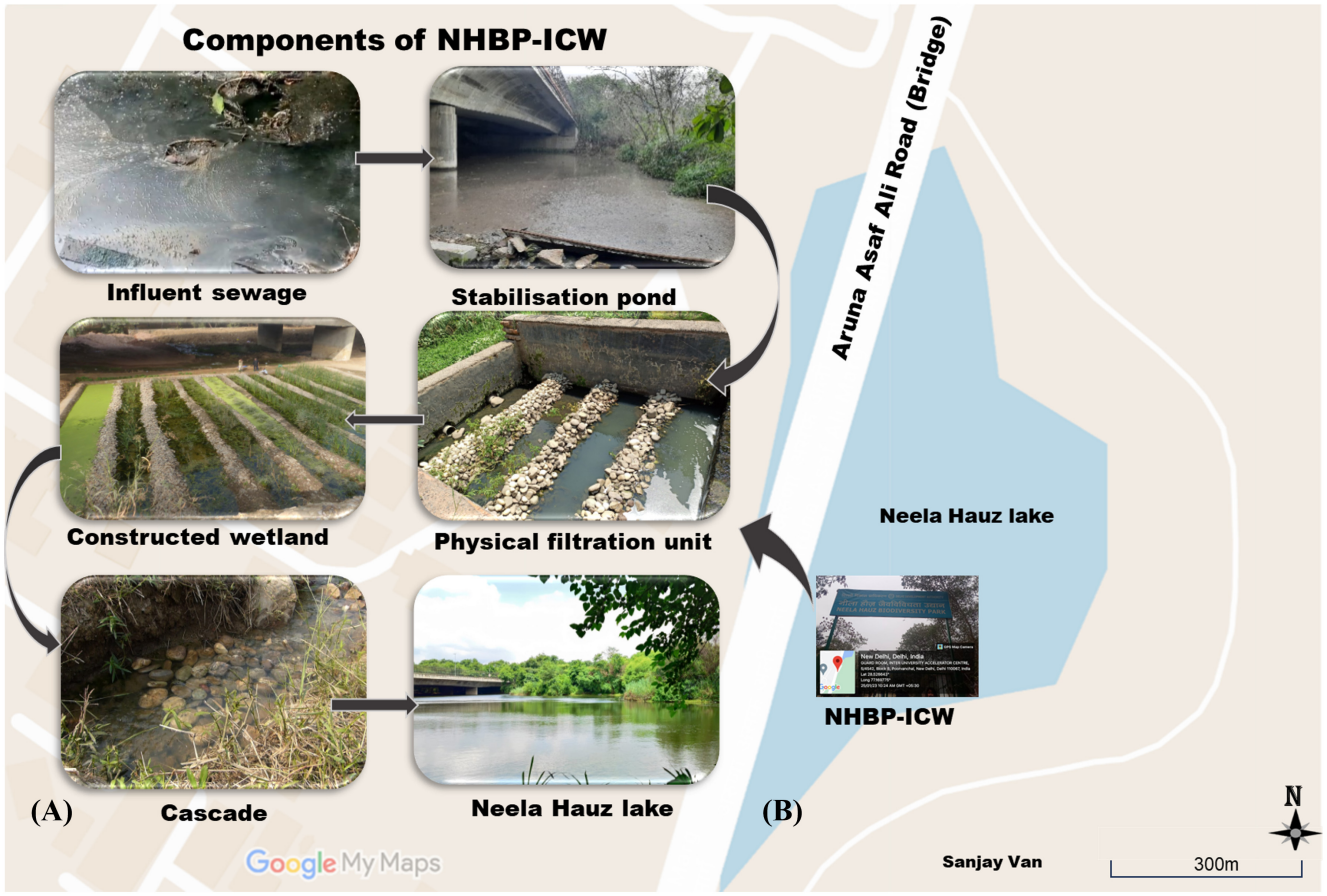
Google map showing various components of the **(A)** Integrated Constructed Wetland in **(B)** Neela Hauz Biodiversity Park (28.53 N 77.17 E), New Delhi, India and Neela Hauz Lake.

A free, water surface-flow type integrated constructed wetland system was developed at Neela Hauz Biodiversity Park (NHBP-ICW), New Delhi, India in 2014 (Jaggi, 2017; Delhi Biodiversity Park Organisation, 2016; www.delhibiodiversityparks.org; Figure 1A). *In situ* remediation of 1 MLD wastewater occurs at NHBP-ICW and it has been functioning for the last 10 years without external energy input (Jaggi, 2017; Delhi Biodiversity Park Organisation, 2016; www.delhibiodiversityparks.org). This integrated constructed wetland system has two stabilization ponds where the influent sewage is retained up to 10–12 h for sedimentation of suspended solids and activation of sludge; a physical filtration unit with rough rock filters to enhance the dissolved oxygen (DO) levels due to turbulence of flowing sewage water through pores; and a vegetated wetland unit (Figure 1B) with around 20 aquatic plant species including *Typha latifolia* L.*, Phragmites karka* (Retz.) Trin. ex Steud.*, Lemna sp., Ipomoea aquatica* Forssk*., Alternanthera philoxeroides* (Mart.) Griseb. with rhizospheric microbes that likely contribute to the perceptible *in situ* bioremediation of sewage water that then flows into the downstream freshwater lake (Jaggi, 2017; Seth et al., 2024b,a). The design of the integrated constructed wetland with a brief account of the microbial communities detected in sludge and sediment and physicochemical parameters from the CW components were reported previously (Seth et al., 2024b,a).

Despite several studies on microbial communities of CWs, the microbial functional diversity associated with biotransformation of sewage wastes is not well understood. To address this knowledge gap, the putative functional diversity associated with sludge samples from a stabilization pond and rhizospheric sediments sampled from vegetation in the constructed wetland unit of the NHBP-ICW (Figure 1) was evaluated using 16S rRNA gene NGS (Next Generation Sequencing) dataset and microbial functional databases. The objectives of this study were to examine the stabilization pond sludge and wetland rhizospheric sediment from NHBP-ICW for differences in bacterial community structure, inferred functions, and association with water quality gradient. The findings of this study not only highlight bacterial taxa and predict functional processes that may be responsible for amelioration of contaminants from influent sewage in NHBP-ICW but also provide new insights into future applications of bacterial communities for enhanced wastewater management in constructed wetland systems.

## Material and methods

### Study site and sample description

The integrated CW developed at Neela Hauz Biodiversity Park (28.53 N 77.17 E), New Delhi, India has two stabilization ponds, a filtration zone and a vegetated CW unit (Figure 1). The details for the development of CW as well as sampling of sediment and sludge have been mentioned in a previous study (Seth et al., 2024b). Sludge samples were collected from one of the stabilization ponds and sediment samples were collected from the rhizospheres of *Typha latifolia* L. and *Phragmites karka* (Retz.) Trin. ex Steud growing in the vegetated wetland. Bacterial communities were targeted using the 16S rRNA gene V3-V4 region metagenomic sequencing using Illumina MiSeq platform (300X2 bp) as described in Seth et al. (2024b). The NGS data were generated for three independent biological replicates representing each sampling site, and is available in NCBI SRA database under BioProject Accession Number PRJNA1052432 (BioSamples from sludge component of the stabilization pond: SAMN38822400, SAMN38822401, SAMN38822402 and BioSamples from rhizospheric sediments of the constructed wetland component: SAMN38822403, SAMN38822404, SAMN38822405). Samples of sludge, sediment and sewage water were collected on the same day, time and place within the NHBP-ICW components. Physico-chemical measurements representing the stabilization pond sludge and rhizospheric wetland sediment samples (from which environmental DNA was isolated for NGS analysis) were conducted as described previously (Seth et al., 2024a) for environmental variables like NH_3_-N, PO_4_-P, BOD, COD, TDS, TSS and pH.

### Identification of differentially abundant bacterial taxa at different taxonomic levels and their putative functional diversity involved in bioremediation of sewage

The raw NGS data were processed using QIIME2 ver. 2021.11 (Bolyen et al., 2019) using prepared manifest file. The reads were denoised and filtered using DADA2 plugin. The Operational Taxonomic Units (OTUs) were clustered at a similarity threshold of 99% using VSEARCH plugin. The taxonomic classification was done using SILVA138 database (Quast et al., 2013). The relative abundance of various taxa associated with stabilization pond sludge and rhizospheric sediments from vegetated wetland were compared using “*microeco*” package in R (R Core Team, 2021). The putative relative abundance of various functional processes was predicted for the NGS dataset using FAPROTAX (Functional Annotation of Prokaryotic Taxa) database v1.2.12 (Louca et al., 2016). It converts OTU tables into functional tables based on taxa identified in a sample and functional phenotype attributed to each taxon in the reference FAPROTAX database. The predicted relative abundance of different microbes involved in processes related to nitrogen respiration, sulphur respiration, and methanogenesis were compared between the two components of the NHBP-ICW. Different bacterial orders and genera in the microbial community were functionally annotated using metabolic information retrieved from different functional databases including MACADAM (MetAboliC pAthways Database) database (Le Boulch et al., 2019) and MiDAS4 (Microbial Database for Activated Sludge) database (Dueholm et al., 2022; https://www.midasfieldguide.org/guide/search).

### Co-occurrence network analyses of associated bacterial taxa and multivariate analyses of microbial communities with water quality

A co-occurrence network based on Spearman's correlation coefficients was constructed (threshold relative abundance > 0.0009, *p*-value = 0.05, optimal correlation cutoff = 0.72) for bacterial OTUs in different samples from NHBP-ICW using “*microeco*”, “*mecodev*” packages in R. Chord plots were generated at different taxonomic levels (Phylum and Order level) using “*circlize*” package in R. The constructed network was imported into Gephi ver 0.10 to compute various network attributes. Bivariate Spearman correlation coefficients were computed between the relative abundances of bacteria (Phylum, Order and Genus level) in different samples and the corresponding sewage quality parameters of the samples. The resulting correlation matrix showing taxa (that are also known to occur in other sewage and CW ecosystems), with significant correlation coefficients in this dataset was then visualized as a heatmap using the “*ggplot2*” package in R. A principal component analysis (PCA) was first conducted using various environmental variables (Supplementary Table 1). Since PC1 captured most of the shared variation across the physicochemical variables (~91%) indicating collinearity, representative variables (NH_3_-N and COD) were selected for subsequent analyses based on their differential loadings on PC2 and highest eigenvalues explaining the total variance (Supplementary Tables 1 and 2). In addition, scores of PC1 were extracted to represent a dominant environmental gradient and used as a composite explanatory variable in a subsequent exploratory analysis (Supplementary Tables 1 and 2). Canonical Correspondence Analyses (CCA) was done using the representative environmental variable(s), along with community composition data at various taxonomic levels (Phylum and Genus levels) using “*vegan*” package in R.

## Results

### Analyses of relative abundance of bacterial taxa at different taxonomic levels

**Figure 2:**
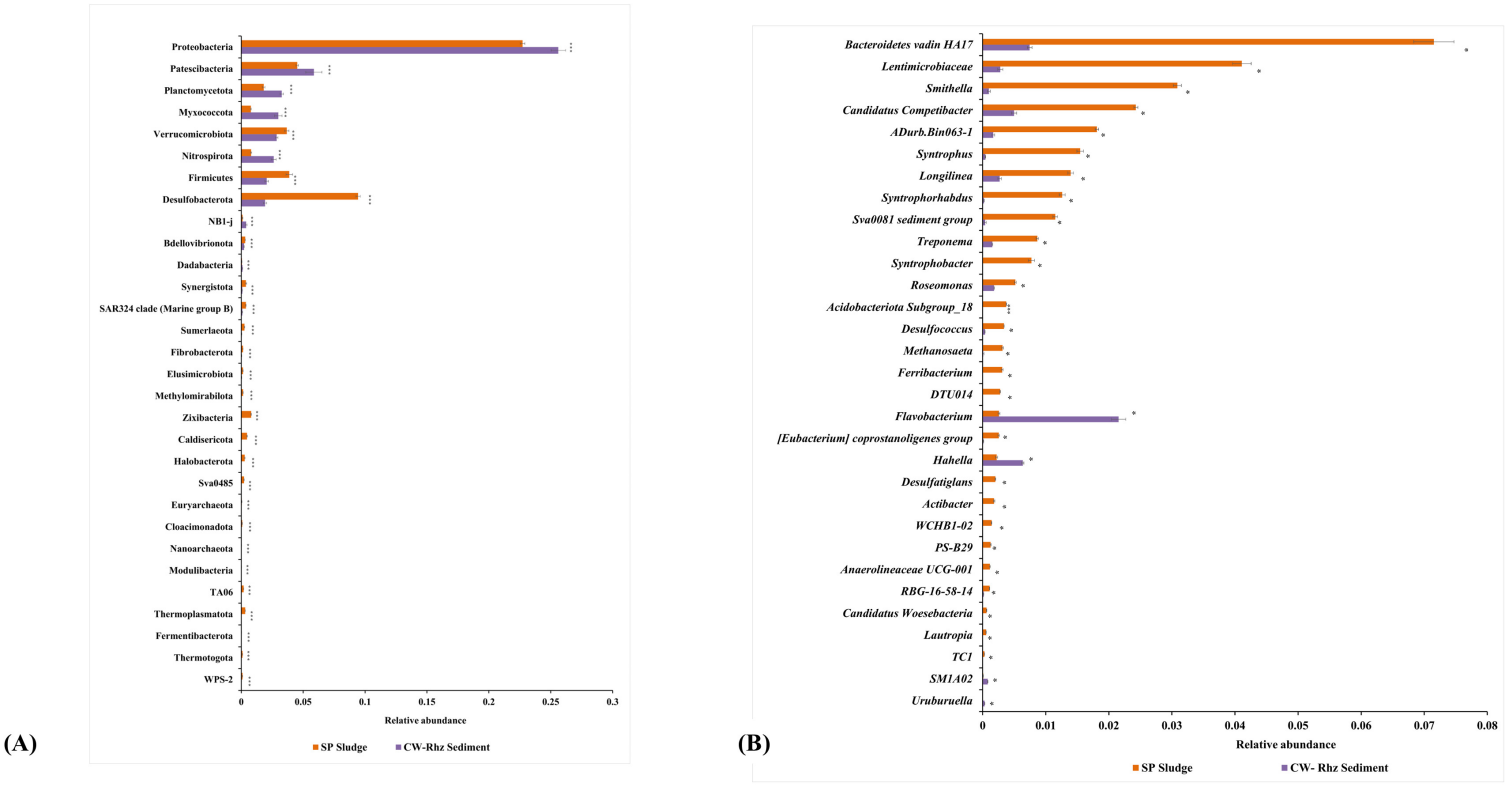
Relative abundance of bacterial **(A)** phyla (*p* < 0.05) and **(B)** genera (*p* < 0.01) showing significant differences between stabilization pond sludge and rhizospheric sediment samples from NHBP-ICW (denoted as *p < 0.05; ***p < 0.001).

Different bacterial phyla and genera with significant differences in their relative abundance between the sludge and sediment microbial community datasets were identified (Figure 2). While the relative abundance of bacterial phyla *Desulfobacterota* (Sludge-0.09 ± 0.01; Sediment-0.02 ± 0.001)*, Spirochaetota* (Sludge-0.022 ± 0.000; Sediment-0.003 ± 0.0001), *Firmicutes* (Sludge- 0.038 ± 0.0002; Sediment −0.02 ± 0.001), was significantly higher (*p* < 0.001) in stabilization pond sludge, the relative abundance of *Nitrospirota* (Sludge-0.001 ± 0.000; Sediment-0.008 ± 0.000)*, Planctomycetota* (Sludge-0.02 ± 0.000; Sediment-0.03 ± 0.000) was significantly (*p* < 0.001) higher in rhizospheric sediments (Figure 2A).

Among bacterial genera, *Smithella* (*Desulfobacterota, Syntrophales*; Sludge-0.031 ± 0.000; Sediment-0.007 ± 0.0003)*, Syntrophus* (*Desulfobacterota, Syntrophales*; Sludge-0.015 ± 0.000; Sediment- 0.0004 ± 0.000)*, Syntrophorhabdus* (*Desulfobacterota, Syntrophorhabdales*; Sludge-0.013 ± 0.000; Sediment-0.0002 ± 0.000)*, Desulfatiglans* (*Desulfobacterota, Desulfatiglandales*; Sludge-0.007 ± 0.000; Sediment-0.002 ± 0.00) showed significantly (*p* < 0.05) higher relative abundance in microbial community associated with stabilization pond (Figure 2B). Contrary to this, *Hahella* (*Proteobacteria, Oceanospirillales*; Sludge-0.002 ± 0.0001; Sediment-0.006 ± 0.0001)*, Denitratisoma* (Proteobacteria, Burkholderiales; Sludge-0.0005 ± 0.000; Sediment-0.001 ± 0.000)*, Flavobacterium* (*Bacteroidota, Flavobacteriales*, Sludge-0.002 ± 0.000; Sediment-0.02 ± 0.000)*, Bifidobacterium* (*Actinobacteriota, Bifidobacteriales*; Sludge-0.0003 ± 0.000; Sediment-0.001 ± 0.000) and *Accumulibacter* (*Proteobacteria, Burkholderiales*; Sludge-0.0001 ± 0.000; Sediment-0.001 ± 0.000) were significantly (*p* < 0.05) more abundant in microbial communities belonging to the rhizospheric sediments (Figure 2B). *Pseudomonas* (*Proteobacteria, Pseudomonadales*) and *Bradyrhizobium* (*Proteobacteria, Rhizobiales*) were found in both stabilization pond sludge and rhizospheric sediment microbial community. Their relative abundance was higher (*p* < 0.05) in the stabilization pond sludge samples as compared to the rhizospheric sediment (Figure 2B). The genus *Nocardioides* (*Actinobacteriota, Propionibacteriales*) was found to be prevalent in sludge microbial community. *Paracoccus (Proteobacteria, Rhodobacteriales*), *Uruburuella* (*Proteobacteria, Burkholderiales*)*, Rhodoplanes (Proteobacteria, Rhizobiales*) and *Brevundimonas* (*Proteobacteria, Caulobacteriales*) were found only in sludge samples and the genus *Rhodobacter* (*Proteobacteria, Rhodobacteriales)* was found only in sediment samples (Figure 2B; Supplementary Figure 1A). Several other genera like *Rhodanobacter* (*Proteobacteria, Xanthomonadales*) and *Dokdonella* (*Proteobacteria, Xanthomonadales*) were only found in sludge while *Thermomonas* (*Proteobacteria, Xanthomonadales*), *Thiothrix* (*Proteobacteria, Thiotrichales*) and *Geobacter (Desulfobacterota, Geobacterales*) were found only in sediment samples (Supplementary Figure 1B). Among the uncharacterised rare bacterial genera, *AKYH767* (*Bacteroidota, Sphingobacteriales)* and *SM1A02* (*Planctomycetota, Phycisphaerales)* were significantly (*p* < 0.05) more prevalent in rhizospheric sediment samples, *BSV13* (*Bacteroidota, Bacteroidales)* and *ST-12K33 (Bacteroidota, Sphingobacteriales)* showed significantly (*p* < 0.05) higher relative abundance in stabilization pond sludge (Supplementary Figure 1B). Further work with larger number of spatiotemporal samplings is needed from contrasting sites of NHBP-ICW to confirm the trends observed in relative abundance of bacterial communities from this study. Nevertheless, the comparison of microbial communities at different taxonomic levels indicated that the bacterial assemblages associated with rhizospheric sediments showed higher diversity as compared to the sewage sludge in stabilization pond.

### Putative functional diversity associated with bacterial taxa involved in bioremediation of sewage

**Figure 3:**
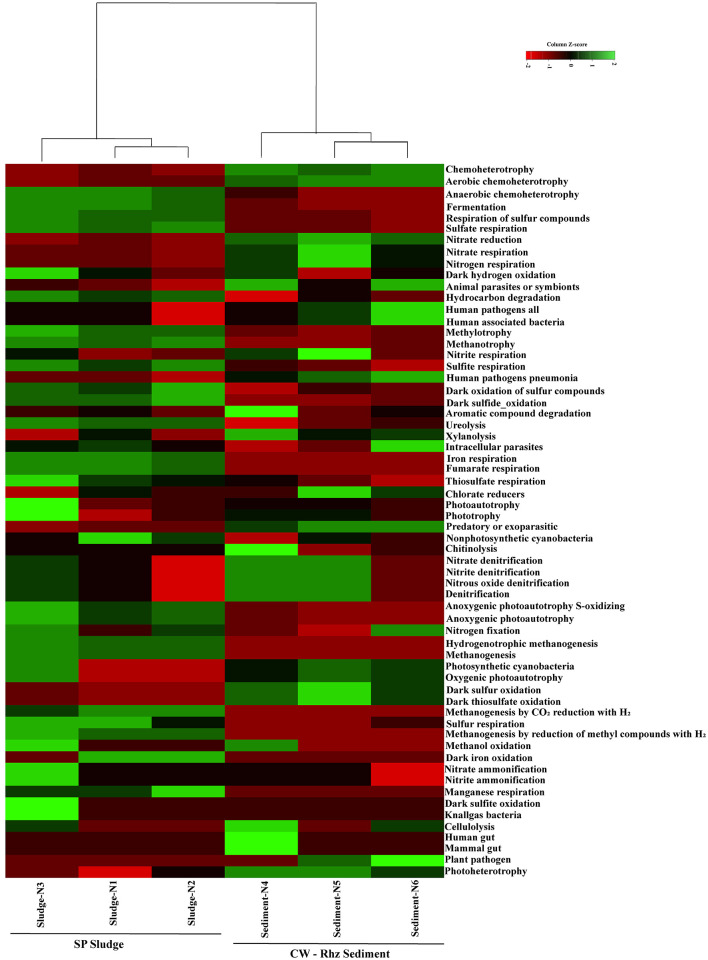
Heatmap showing z-score values for the predicted abundance of functional groups in different samples predicted on the basis of FAPROTAX database.

**Figure 4:**
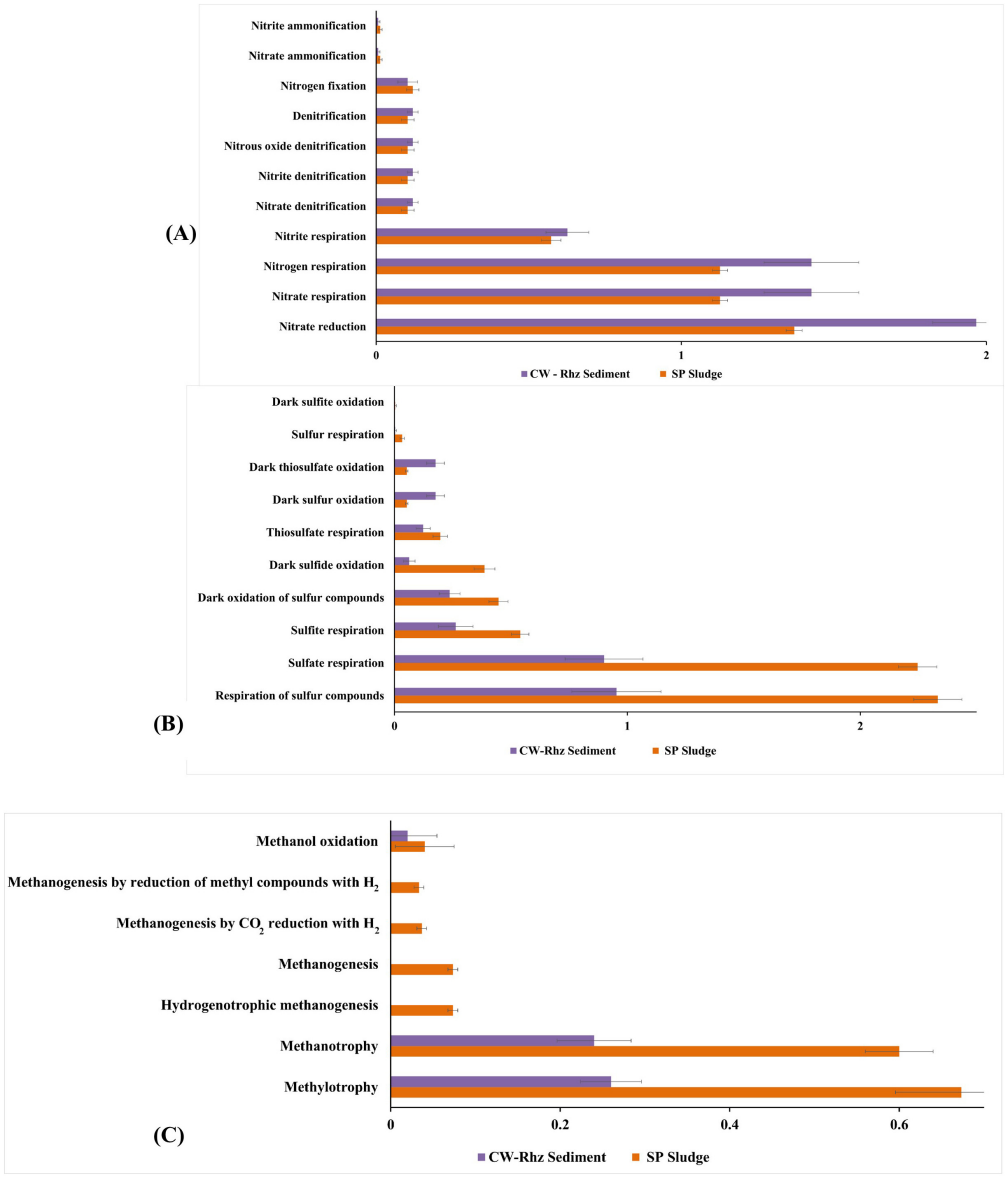
Predicted relative abundance of bacteria involved in different microbial functional processes related to **(A)** Nitrogen respiration, **(B)** Sulphur respiration and **(C)** Methanogenesis observed in stabilization pond sludge and rhizospheric sediment samples from vegetated wetland unit in NHBP-ICW.

Different functional processes were predicted for the microbial communities from the stabilization pond and vegetated constructed wetland at NHBP-ICW (Figure 3). Predictions that were distinct among the metagenomes from two sites are described here. Among energetic reactions, anaerobic chemoheterotrophy was likely to be prevalent in microbes from the stabilization pond samples, while the abundance of microbes predicted to be involved in aerobic chemoheterotrophy was seen to be higher in rhizospheric sediment samples (Figure 3). Figure 3 also indicated the likely occurrence of microbial processes participating in biogeochemical cycles and transformation of different elements, including carbon fermentation (in C-Cycle), sulphate respiration (in S-Cycle) and nitrate respiration (in N-Cycle) in both the samples. Elevated relative abundance of microbes with putative functions related to nitrogen reduction such as nitrate reduction, nitrite reduction in the rhizospheric sediments also suggested a predominance of aerobic processes (Figure 4A). Microbial processes linked to sulfate, sulfite respiration and thiosulphate oxidation were predicted to be enriched in stabilization pond sludge as compared to the rhizospheric sediment samples (Figure 4B). Unlike the bacterial assemblages in the rhizospheric sediments, various methanogenic processes involving reduction of carbon dioxide and methyl compounds were likely to be more prevalent in the stabilization pond sludge microbial community (Figure 4C). Other inferred processes included methylotrophy and methanotrophy associated with microbes that were abundant in the sludge microbial community from the stabilization pond. Overall, the predicted functional profile from the NHBP-ICW NGS dataset suggested the occurrence of distinct microbial metabolic potential across the two constructed wetland components: stabilization pond sludge community being dominated by microbes that can perform anaerobic carbon and sulfur cycling processes along with methane producing processes, while the bacterial community associated with sediment samples was likely to be enriched in bacteria that supported aerobic nitrogen and carbon oxidation processes. These results, albeit predictive, nevertheless provide testable hypotheses that can be validated downstream with experiments related to bacterial heterotrophy, biogeochemical cycles, organic matter turn-over and pollutant break-down.

### Potential associations of bacterial taxa/groups in sludge and sediment from the NHBP-ICW ecosystem

**Figure 5:**
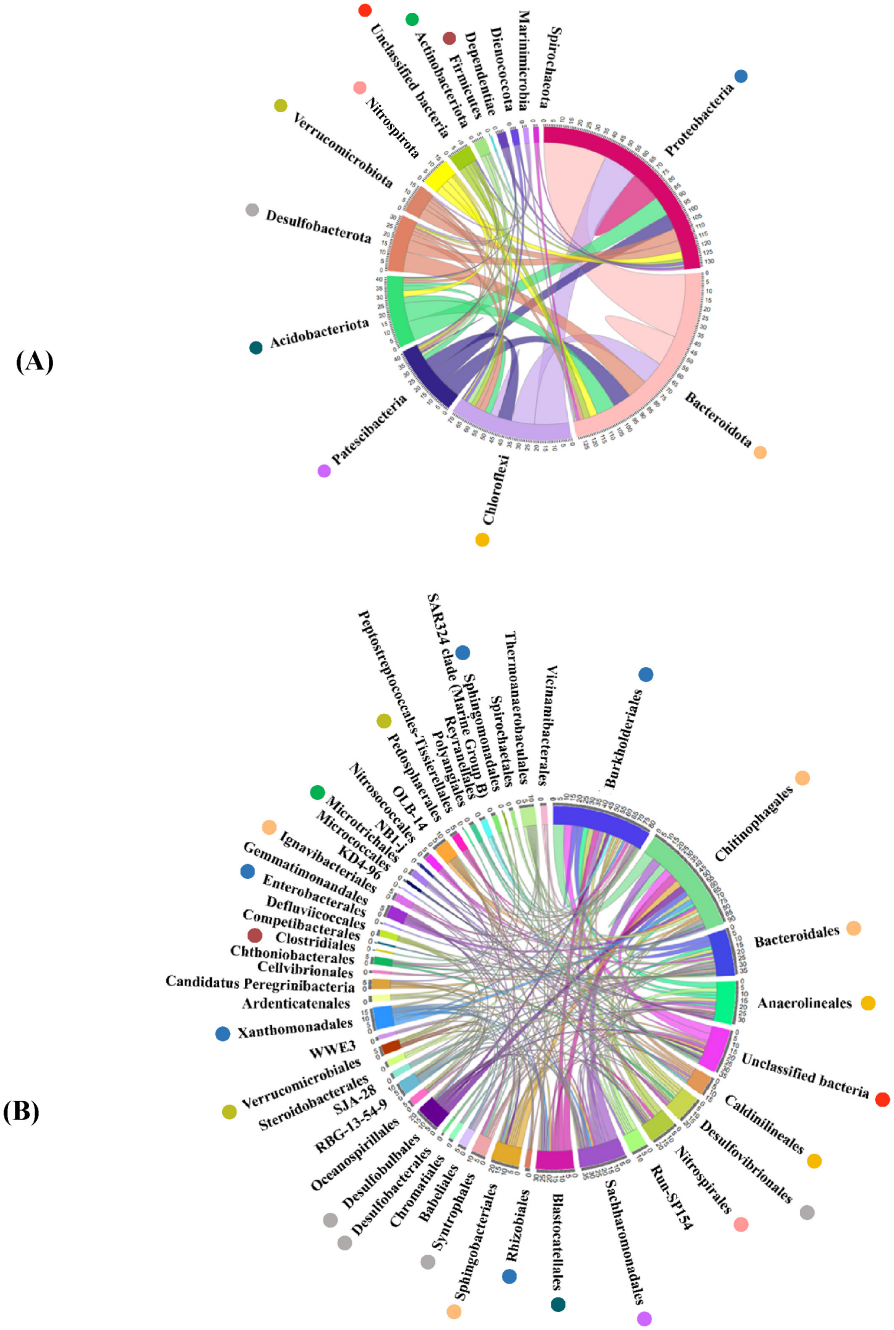
Chord plot representation of the co-occurrence correlation network of bacterial taxa at **(A)** phylum level and **(B)** order level. The network was based on Spearman correlations (*p*-value = 0.05, correlation cutoff = 0.72, OTU R.A. threshold > 0.0009) constructed using microeco package in R and Gephi. Bacterial orders belonging to a phylum are denoted with circles of the same color.

A co-occurrence network was constructed based on the Spearman's correlation coefficients (OTU threshold relative abundance > 0.0009, *p*-value = 0.05, correlation cutoff = 0.72) computed for the OTUs analyzed at phylum and order level in the dataset (Figure 5). Bacterial taxa from *Proteobacteria* and *Bacteroidota* were seen to form the maximum number of associations followed by *Chloroflexi, Patescibacteria, Acidobacteriota* and *Desulfobacterota* in the network at the phylum level (Figure 5A). This dataset also showed that members belonging to *Nitrospirota* formed associations with members of *Proteobacteria, Bacteroidota* and *Acidobacteriota*. Bacteria belonging to *Marinimicrobia* were seen to form association with bacteria belonging to *Desulfobacterota* and *Verrucomicrobiota* along with *Bacteroidota*. Members of the phylum *Spirochaetota* were seen to have associations with members of *Bacteroidota* and *Proteobacteria*. Interestingly, this dataset also showed that unclassified bacterial sequences were associated with several prevalent phyla including *Proteobacteria, Bacteroidota, Chloroflexi, Desulfobacterota, Patescibacteria* and *Acidobacteriota* (Figure 5A).

At the order level, the network based upon this NGS dataset highlighted potential co-occurrence association patterns of various bacterial orders within and across different phyla (Figure 5B). *Chitinophagales* from *Bacteroidota* were seen to form the highest number of associations (28) with the other bacterial orders including *Burkholderiales (Proteobacteria), Caldilineales* (*Chloroflexi*)*, Xanthomonadales* (*Proteobacteria*)*, Nitrospirales* (*Nitrospirota*)*, Microtrichales* (*Actinobacteriota*) and *Sphingobacteriales (Bacteroidota)*. Following this, *Burkholderiales* were seen to form the second highest number (15) of associations with *Chitinophagales, Bacteroidales, Blastocatellales, Anaerolineales, Ignavibacteriales* and *Pedosphaerales* along with *Xanthomonadales* and *Syntrophales*. Bacterial orders like *Desulfobulbales, Desulfobacterales, Desulfovibrionales* belonging to the phylum *Desulfobacterota* were found to form associations with *Chitinophagales* and *Bacteroidales* from *Bacteroidota* along with *Anaerolineales* belonging to *Chloroflexi* in this dataset (Figure 5B). Similar to the network analyzed at phylum level, several orders were also observed to form associations with unclassified bacterial sequences. Further work with increased, longitudinal sampling effort is needed across the CW ecosystem to assess dynamics of microbial associations identified in this study.

### Correlation analyses predict association of bacterial groups with sewage quality parameters in NHBP-ICW

**Figure 6:**
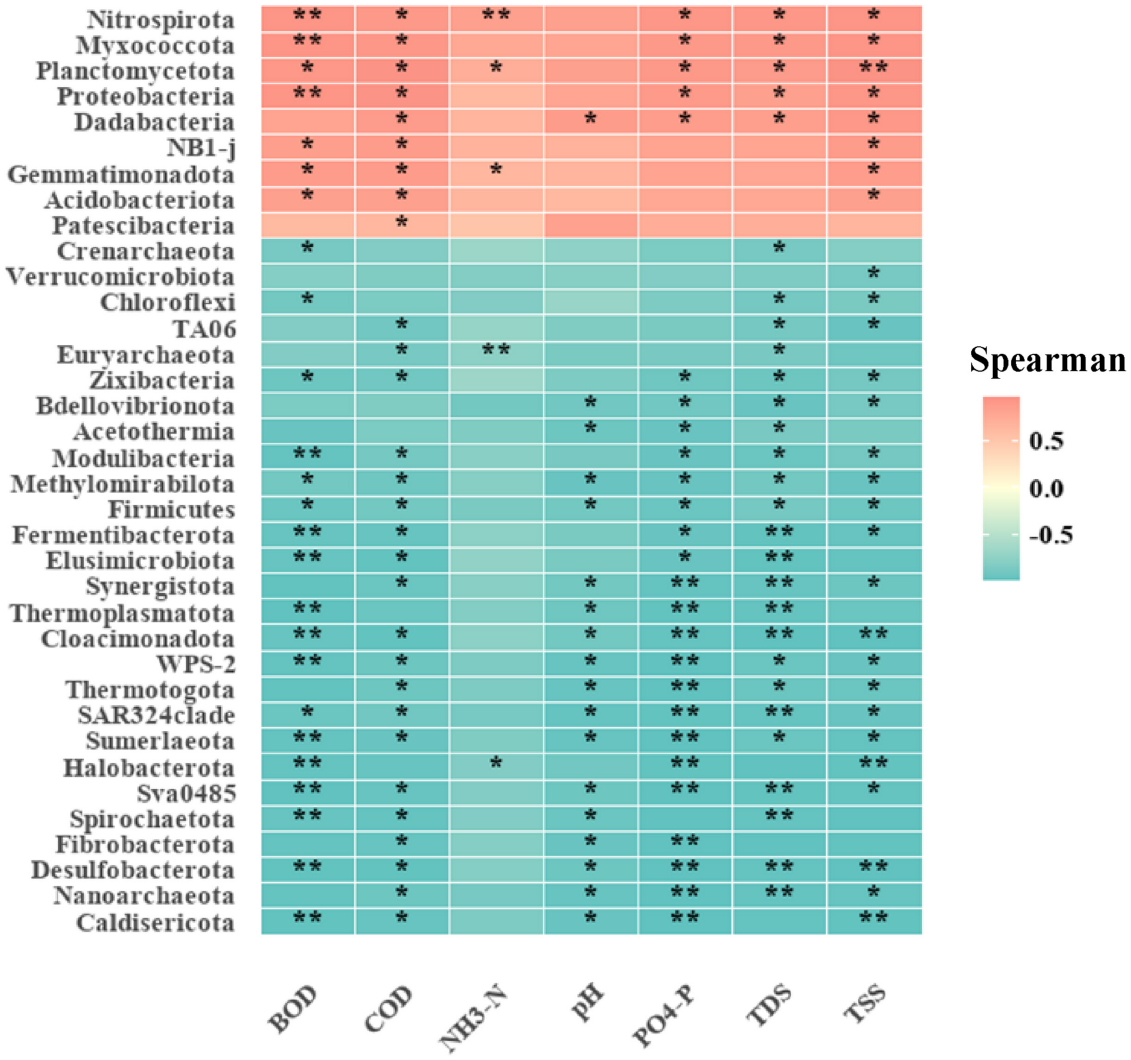
Heatmap showing Spearman correlation coefficient computed for bacterial phyla with different sewage water quality parameters. Bacterial phyla with significant correlations with at least one of the sewage water quality parameters are shown here (denoted as: * *p* < 0.05; ** *p* < 0.01).

Correlation analysis suggested that several bacteria across phylum, order, and genus levels from this dataset may be associated with multiple physicochemical parameters, reflecting selective responses to prevailing environmental gradient (Figure 6). At the phylum level, members from this dataset comprising *Acidobacteriota, Gemmatimonadota, Myxococcota, Proteobacteria, Planctomycetota, Nitrospirota* and *NB1-j* were seen to be associated (*r* > 0.85; *p* < 0.05) with changes in levels of COD, BOD and TSS (Figure 6). *Dadabacteria* was seen associated (*r* > 0.85; *p* < 0.05) with COD, pH, TDS, TSS and PO_4_-P (Figure 6). Other phyla such as *Myxococcota, Nitrospirota, Proteobacteria, Planctomycetota* showed similar associations (*r* > 0.80; *p* < 0.05) with TDS and PO_4_-P levels (Figure 6). Few bacterial phyla including *Nitrospirota* (*r* > 0.85; *p* < 0.001), *Gemmatimonadota* and *Planctomycetota* (*r* > 0.85; *p* < 0.05) were associated with the changes in levels of NH_3_-N (Figure 6).

At the order level, *Chitinophagales (Bacteroidota), Dadabacteriales (Dadabacteria), Xanthomonadales (Proteobacteria), Planctomycetales (Planctomycetota), Bifidobacteriales (Actinobacteriota)* and *Saccharimonadales (Patescibacteria)* from this dataset were found to be associated with PO_4_-P (Supplementary Figure 2A). *Flavobacteriales, Xanthomonadales, Bifidobacteriales* and *Saccharimonadales* were likely to be associated with TDS. Members of *Planctomycetales* and *Streptomycetales (Actinobacteriota)* also showed associations with changes in TDS and other physicochemical parameters like BOD and TSS (Supplementary Figure 2A). Several low abundance, uncharacterised bacterial orders like *OM190 (Planctomycetota), JG36-TzT-191 (Proteobacteria), Run-SP154 (Proteobacteria)* belonging to major bacterial phyla in the community were also likely to be associated with changes in various physicochemical parameters like BOD, COD, TSS, TDS, pH as well as PO_4_-P (*r* > 0.75; *p* < 0.05).

Several genera from the above phyla including *Nitrosomonas (Proteobacteria, Burkholderiales), Accumulibacter (Proteobacteria, Burkholderiales), Hahella (Proteobacteria, Oceanospirillales), Hydrogenophaga (Proteobacteria, Burkholderiales), Unclassified Blastocatellales, Unclassified Saccharimonadales (Patescibacteria), Unclassified Planctomycetales (Planctomycetota)* in this dataset demonstrated association (*r* > 0.90; *p* < 0.05) with multiple parameters such as BOD, COD, TDS, TSS, pH and PO_4_-P (Supplementary Figure 2B). *Unclassified Planctomycetales (Planctomycetota)* was also one of the few genera that showed an association with levels of NH_3_-N (*r* > 0.70; *p* < 0.01; Supplementary Figure 2B). Despite the limited sampling, several uncultured and uncharacterised taxa were seen (*r* ≥ 0.90; *p* < 0.05) associated with sewage quality parameters including *SH3-11 (Verrucomicrobiota, Pedosphaerales), OM190 (Planctomycetota), JG36-TzT-191 (Proteobacteria)*, and *SM1A02 (Planctomycetota, Phycisphaerales)* which may indicate unique associations with novel functions in removal of wastewater pollutants. Further work is needed to test hypotheses that validate the inferred microbial associations from this study with the ICW environment.

### Relationship between water quality parameters and microbial community composition and structure

Sewage-water is the primary source of microbial communities besides rhizospheric microbial communities of aquatic plants introduced into the vegetated wetland unit of NHBP-ICW. The microbial communities interact when the sewage water is retained in both stabilization pond and vegetated wetland, presumably leading to altered water quality and/or removal/degradation of pollutants. An important deliverable of research on the NHBP-ICW is to ultimately predict the relationship between the microbial community diversity of the stabilization pond sludge component and vegetated wetland sediment component by correlating the total percent removal efficiency of the pollutants (calculated from the values in the inlet/raw sewage and the final outlet from vegetated wetland) with the relative abundance of bacteria in microbial communities as revealed by this metagenomic snapshot from contrasting sites in NHBP-ICW.

**Figure 7:**
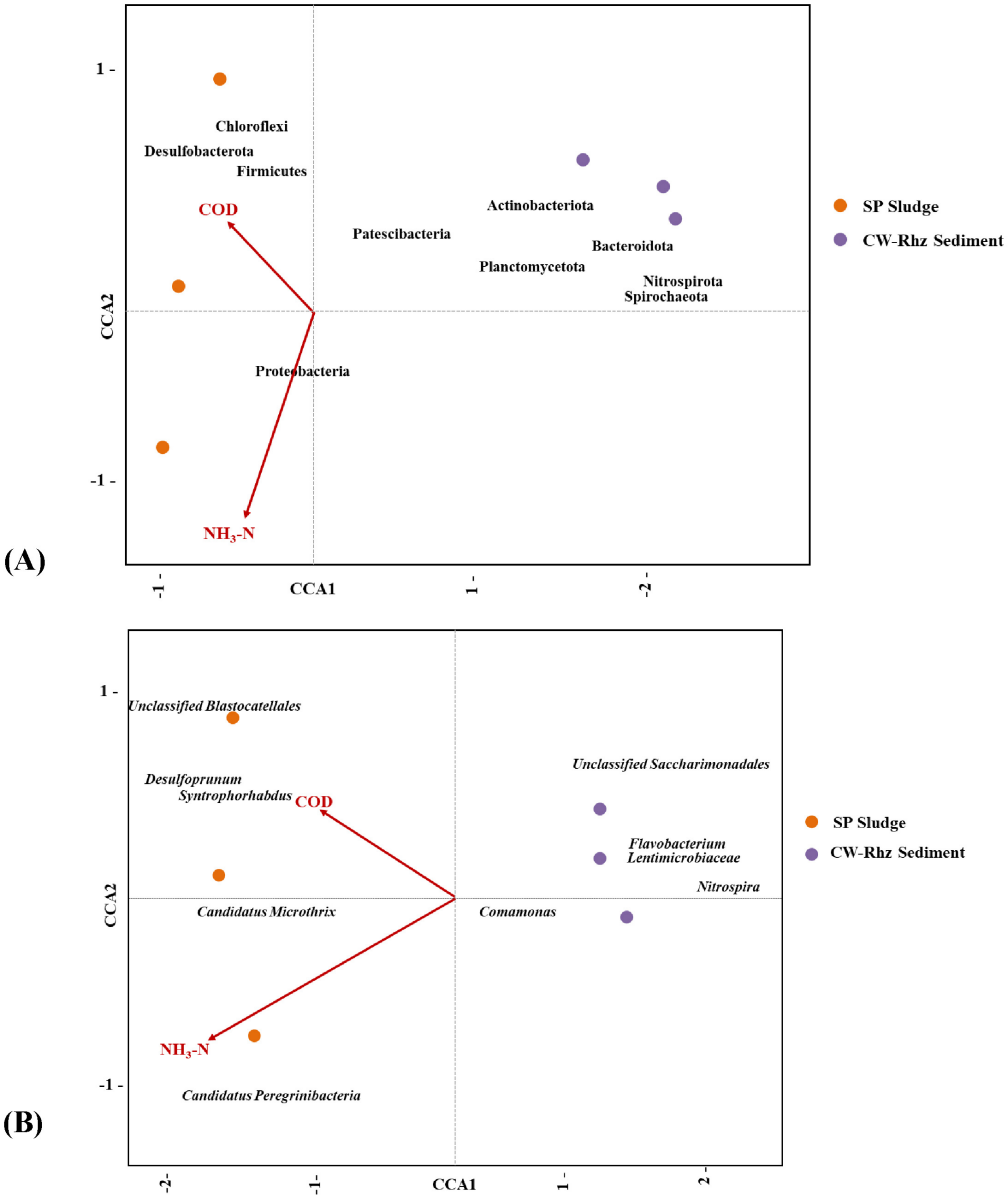
Results of CCA for microbial community composition at **(A)** phylum and **(B)** genera levels with physicochemical parameters for stabilization pond sludge and rhizospheric sediment samples (Top 10 taxa).

Different physicochemical parameters including NH_3_-N, COD, TSS, PO_4_-P, TDS, BOD, and pH showed lowered values in rhizospheric sediment in comparison to stabilization pond sludge forming an environmental gradient (Supplementary Figure 3). Among the correlated physicochemical parameters, two representative variables NH_3_-N and COD were used for further analysis based upon their loadings on PC2 (Supplementary Tables 1, 2). NH_3_-N with the highest eigenvalue (0.858) explained 42.9% of the total variance, followed by COD which explained 15.7% of the total variance (Supplementary Table 2). A CCA ordination showed that microbial communities in the rhizospheric sediment samples were well separated from the stabilization pond sludge samples (Figure 7A). Bacterial phyla like *Chloroflexi, Desulfobacterota* and *Firmicutes* were seen proximal to stabilization pond sludge samples while *Actinobacteriota, Bacteroidota, Planctomycetota, Patescibacteria* and *Nitrospirota* were seen proximal to the constructed wetland rhizospheric sediment samples (Figure 7A). Similar results were seen at the genus level with the sludge and sediment samples being well separated from each other (Figure 7B). The CCA plot showed bacterial genera like *Desulfoprunum, Candidatus Peregrinibacteria, Candidatus Microthrix*, and *Syntrophorhabdus* clustered near stabilization pond sludge samples (Figure 7B). On the other hand, *Nitrospira, Comamonas, Leptolinea, Unclassified Saccharimonadales*, and *Flavobacterium* were seen in the proximity of the rhizospheric sediment samples (Figure 7B). Similar results were obtained from a secondary sensitivity analysis to assess the robustness of the ordination (Supplementary Figure 4). The composite environmental gradient accounted for 84.71% of the variation in microbial community composition at the phyla level (Supplementary Figure 4). While many bacterial phyla showed relatively weak responses to the gradient, *Chloroflexi, Desulfobacterota*, and *Spirochaetota* were observed proximal to the sludge samples while *Actinobacteriota, Nitrospirota* were seen proximal to the sediment samples (Supplementary Figure 4) as also noted in Figure 7. Similar trends were observed at the genus level, with the sludge and sediment communities being distinct along the environmental gradient which explained 77.90% of the variation in community composition (Supplementary Figure 4).

However, it must be mentioned that given the limited sample size, these findings should be interpreted as exploratory, reflecting variation in distribution of bacterial groups across samples or components of NHBP-ICW. Further work with improved sampling strategies that critically examine the microbial associations identified in this study and their relation to environmental gradients across distinct sites of CW ecosystem and identify bacterial functional groups that likely contribute to amelioration of sewage-rich environments.

## Discussion

Applications of metagenomic sequencing methods have contributed to the understanding of bacterial community structure, and their inferred metabolic potential within different components of constructed wetland systems (Kurzbaum et al., 2012; Kushwaha et al., 2024). Constructed wetland management with continuous assessment of microbial communities and fluxes in environmental parameters can help in monitoring the spatial succession of specialized microbial functional groups such as nitrifying bacteria, sulphur reducing bacteria or bacterial groups carrying out transformation of organic matter along with their dynamics with various sewage quality parameters. These assessments allow for adaptive management practices that optimize and /or enhance the CW performance and ensure the long-term stability of the wetland ecosystems (Malyan et al., 2021; Tang et al., 2021). This study characterizes the complex functional diversity predicted for the bacterial communities sampled from the two components of a decadal, fully operational NHBP-ICW (Figure 1): a stabilization pond sludge and rhizospheric sediments from aquatic plants in the constructed wetland involved in removal of pollutants and bioremediation of sewage. Stabilization ponds play a significant role in constructed wetland systems in biodegradation of pollutants, including reduction in coliform density by sedimentation of suspended particulate matter and activation of the sludge to reduce the pollutant load before the sewage water enters the physical filtration unit or the vegetated wetlands and thereby enhancing the overall efficiency and sustainability of the treatment process (Tsalkatidou et al., 2009; Kurzbaum et al., 2012). Estimates of the sewage quality parameters such as NH_3_-N, PO_4_-P, TDS, TSS, COD, BOD and/or pH are often similar in constructed wetland systems including this NHBP-ICW involved in treatment of sewage (Wang et al., 2022; Ali et al., 2024; Seth et al., 2024a). As such, the performance of the integrated CWs is markedly higher with stabilization ponds (Tsalkatidou et al., 2009; Kurzbaum et al., 2012; Seth et al., 2024a).

In stabilization pond, the upper photic layers are exposed to air and sunlight, the deeper layers and settled sludge are characterized by anoxic conditions (Bryant, 1977; Kilian et al., 2024). Likewise, rhizospheres of aquatic species associated with wetland vegetation including *Typha* and *Phragmites* are known to substantially contribute to pollutant removal in constructed wetlands, characterized by complex interactions between the plants and their associated microbial partners (Pietrangelo et al., 2018; Bhargawa et al., 2025). These macrophytes release oxygen into the rhizosphere to create aerobic micro-niches within the typically anoxic wetland system that facilitate aerobic chemoheterotrophic processes (Hernandez-del Amo et al., 2020). Moreover, the plant exudates in the rhizosphere include primary carbon sources, organic compounds and signaling molecules, which influence the structure, composition and functions of the associated microbial communities (Pietrangelo et al., 2018; Hernandez-del Amo et al., 2020). As also observed in this study (Figure 2, Supplementary Figure 1), a higher microbial diversity in sediments is also reported in literature and this high diversity is attributed to enhanced natural biodegradation (Hernandez-Raquet et al., 2013; Liu et al., 2022).

The bacterial community composition was estimated for three replicates of both sludge and sediment samples from the NHBP-ICW. This was done to account for the heterogeneity in the microbial load between biological replicates existing under similar conditions (Bharti and Grimm, 2021). Significant differences were observed at various taxonomic levels in the microbial community composition of the sludge and sediment samples from NHBP-ICW (Figure 2). The differences in relative abundance of bacterial taxa may influence functional processes involved in bioremediation of sewage. For instance, bacterial phylum *Desulfobacterota* has taxa involved mainly in sulphur respiration and sulphate reduction (Murphy et al., 2021). This phylum was significantly more abundant in stabilization pond sludge and is known to drive simultaneous sulfate reduction and metal removal as well as acidity neutralization (Chen et al., 2024). Likewise, phyla *Planctomycetota* and *Gemmatimonadota*, which were abundant in rhizospheric sediment of the NHBP-ICW, are known to facilitate the regulation of anammox and potentially reduce the production of global warming gas nitrous oxide (Van Teeseling et al., 2015; Van Den Berg et al., 2017). The phylum *Nitrospirota*, known for participating in nitrification and commamox processes in aquatic and terrestrial ecosystems, was abundant in rhizospheric sediments (Van Den Berg et al., 2017).

*Myxococcota*, which was observed in all the samples in this study, is reported to thrive in organic matter and is known to have predatory roles. It is also known to produce various antifungal, antimicrobial secondary metabolites (Saggu et al., 2023). Other bacterial phyla including *Acidobacteriota, Bacteroidota* and *Patescibacteria* prevalent in rhizospheric sediment samples are known to be involved in fermentation and degradation of organic and inorganic carbon sources (Belova et al., 2018; Herrmann et al., 2019). *Firmicutes* include bacteria which are previously reported from human gut and are involved in carbon fermentation (Belova et al., 2018). These were prevalent in stabilization pond sludge (Figure 2). The taxa within *Chloroflexi* are known to be involved in degradation of hydrocarbons, organic compounds and carbon fermentation (Campbell et al., 2014). These were found to be abundant in stabilization pond sludge as also reflected in the CCA analyses (Figures 2 and 7A).

*Dadabacteria* was found to be prevalent in sediment samples (Figure 2). This phylum is known to degrade organic matter, specifically microbial peptidoglycans and phospholipids (Begmatov et al., 2021). *Actinobacteriota* is reported to contribute to carbon cycling and is considered crucial for nitrification and denitrification in wastewater treatment (Van Teeseling et al., 2015; Van Den Berg et al., 2017). Interestingly, the abundance of these bacterial phyla was also influenced by changes in levels of BOD, COD, TDS, TSS and PO_4_-P (Figure 6), akin to reports in the literature (Wang et al., 2021, 2022; Ali et al., 2024), underscoring their possible roles in amelioration of sewage quality parameters in NHBP-ICW.

Several bacterial genera belonging to *Desulfobacterota (Syntrophales, Syntrophorhabdales), Proteobacteria* (*Xanthomonadales, Geobacterales, Oceanospirillales), Bacteroidota (Flavobacteriales)* and *Actinobacteriota (Bifidobacteriales)* showed significant differences in their relative abundance between the stabilization pond sludge and rhizospheric sediment samples and showed relation/association with sewage quality parameters (Figures 2 and 6, Supplementary Figure 2). For instance, genera which are known for their role in nitrite oxidation (Aguilar et al., 2019; Ajibade et al., 2021), like *Rhodanobacter* and *Dokdonella* were found only in sludge while *Thermomonas, Thiothrix* and *Geobacter* were found only in sediment samples (Supplementary Figure 1A). The genus *Brevundimonas* implicated in solubilisation of tri-calcium phosphate and the phosphorus cycle (Wu et al., 2020), was found only in sludge samples in this study (Supplementary Figure 1A). *Pseudomonas* was found in both sludge and sediment (Supplementary Figure 1A) and is involved in Dissimilatory Nitrate Reduction to Ammonium (Van Den Berg et al., 2017; Wang et al., 2022). *Paracoccus*, known to be a Phosphate Solubilising Bacteria, was found in both sludge and sediment communities in this study (Wang et al., 2022; Van Den Berg et al., 2017).

Besides the removal of nitrogen and phosphorus, genera within CW microbial community (including this study) are usually implicated in removal of emergent pollutants like pharmaceuticals, chemicals, antibiotics and drugs along with metal contaminants (Santos et al., 2019; Lu et al., 2021; Wang et al., 2022). Further work is needed to ascertain the roles of these bacteria in the NHBP-ICW. For instance, *Geobacter*, which was specific to sediment samples in this study (Supplementary Figure 1B), is known to be involved in sulfadiazine (SDZ) biodegradation (Wang et al., 2022). Bacterial genera known to biodegrade tetracycline such as *Novosphingobium, Stenotrophomonas* and *Pseudomonas* (Santos et al., 2019; Lu et al., 2021), were found in both sludge and sediment samples from NHBP-ICW (Supplementary Figure 1B). *Pseudoxanthomonas*, which was also detected in both sediment and sludge (Supplementary Figure 1B), is known for ampicillin degradation (Santos et al., 2019; Zheng et al., 2021). The genus *Nocardioides*, which was prevalent in the sludge microbial community (Supplementary Figure 1B), is well-known for its ability to degrade persistent pollutants (Ma et al., 2023).

The nitrifying bacteria like *Nitrosomonas* and *Nitrospira* are known to be involved in active nitrogen cycling and removal processes in sewage and sludge environments (ElNaker et al., 2020; Wang et al., 2022). *Accumulibacter*, a polyphosphate-accumulating organism, known for its established function in biological phosphorus removal from literature (Wang et al., 2022). This genus was observed to be associated with changes in levels of wastewater, PO_4_-P (Supplementary Figure 2B). While *Hahella* is reported to be associated with marine environments and known to produce diverse secondary metabolites (He et al., 2022), its associations with physicochemical parameters in this study suggested its contribution to the overall bioremediation processes within the constructed wetland (Supplementary Figure 2B). The genus *Hydrogenophaga*, which was also associated with sewage quality parameters (Supplementary Figure 2B), is known to be involved in heterotrophic nitrification and aerobic denitrification, contributing to efficient removal of nitrogen compounds along with oxidation of other elements such as Arsenic (Alvi et al., 2023). The associations observed for rare uncharacterized taxa (*OM190, JG36-TzT-191, SH3-11, SM1A02*) suggest that uncultured and uncharacterized taxa may possess specialized metabolic capabilities crucial for novel functions in wastewater pollutant removal. Similar results are seen in Rochera et al. (2024) and Zhang et al. (2024).

Identification of putative functional roles of bacterial communities from two distinct components of NHBP-ICW (Figures 3 and 4) can provide important baseline information on microbial processes ameliorating water quality in the sewage-dominated environments. Similar functional profiles have been previously attributed to members of bacterial communities based on 16S rRNA gene data obtained from wetlands and soil ecosystem (Sansupa et al., 2021; Ali et al., 2024). Microbial functions that enable temporal changes in water quality, are typically driven by localized physicochemical conditions (Zahui et al., 2021; Colette et al., 2023). Based upon profiles of predicted microbial processes, anaerobic chemoheterotrophy was implied for the sludge samples from the stabilization pond, while aerobic chemoheterotrophy was indicated for microbial communities from the rhizospheric sediments of the constructed wetland (Figure 3). A typical feature of sewage sludge is the prevalence of anaerobic microbial processes driven by predominantly anoxic conditions and high organic pollutant loads (Tsalkatidou et al., 2009; Kurzbaum et al., 2012). This study also predicts association of microbial communities in the NHBP-ICW sludge samples with anaerobic, chemoheterotrophic processes like fermentation, sulfidogenesis and methanogenesis (Figures 3 and 4) that are implicated in breakdown of complex organic materials in sewage and wastewater (Bryant, 1977; Nyieku et al., 2020; Kilian et al., 2024). Methanogenesis by reduction of carbon dioxide and methyl compounds is another characteristic process in highly anoxic sludge environments that can lead to production of greenhouse gas (Nyieku et al., 2020; Zahui et al., 2021). Further work is necessary to explore if members of *Desulfobacterota*, an abundant sulphate reducing phylum in the NHBP-sludge (Figure 2A), can display metabolic dexterity attributed to the ability to utilize a variety of precursors like acetate, lactate, alcohols along with sulphates (Plugge et al., 2011). This study predicted the preponderance of aerobic, chemoheterotrophic processes in the rhizospheric sediment samples from NHBP-ICW samples (Figures 3 and 4). Aerobic micro-niches around wetland rhizospheres are characterized by microbes participating in nitrogen reduction processes, which are indispensable for removal of excess nitrates from the wastewater (Tang et al., 2020; Colette et al., 2023). In addition, the metabolically active environment of hydrophytes used in constructed wetlands are also enriched with plant exudates that harbor bacterial taxa belonging to *Actinobacteriota, Acidobacteriota* and *Bacteroidota* that are capable of efficient organic matter degradation and pollutant transformation (Pietrangelo et al., 2018; Wang et al., 2022). Further work is necessary to explore the role of pertinent bacterial groups in members of *Nitrospirota, Planctomycetota* that were abundant (Figure 2) in the rhizospheric sediment samples from NHBP-ICW.

Predictions of putative microbial functional processes with respect to the particular environmental conditions can enable efficient designing and operation of CWs by facilitating desired microbial processes in specific zones of the CW (Gaikwad et al., 2014; Alabiso et al., 2023; Kushwaha et al., 2024). Identification of microbial taxa associated with changes in undesirable levels of BOD, COD, TSS, TDS, and PO_4_-P in CW is also critical for development of targeted microbial consortia to enhance the removal efficiencies for specific pollutants (Gaikwad et al., 2014; Alabiso et al., 2023; Kushwaha et al., 2024). Hence, putative associations identified among various groups in the NHBP-ICW bacterial community (Figure 5) need to be validated. For example, CW are rich in organic matter and form “excess carbon” environments; that are typically associated with bacterial groups involved in degradation of organic matter and fermentation of carbon products (Zhang et al., 2025). Bacterial orders within *Desulfobacterota* such as *Desulfobulbales, Desulfobacterales, Desulfovibrionales, Syntrophales* are known to be sulphate reducing bacteria (Murphy et al., 2021). In this study, these bacterial orders formed associations with orders such as *Chitinophagales, Blastocatellales, Anaerolineales* within *Bacteroidota, Chloroflexi, Patescibacteria* and *Acidobacteriota* (Figure 5B). Similar co-occurrence of sulphate reducing bacteria with carbon fermenting bacteria has been reported previously in literature as carbon fermentation products can serve as electron donors in sulphur-limiting conditions (Plugge et al., 2011). Other associations of carbon metabolizing bacteria were observed with bacterial taxa involved in nitrate reduction and nitrification (Figure 5). For example, *Nitrospirae* formed associations with different bacterial phyla that include carbon metabolizing taxa including *Proteobacteria, Bacteroidota* and *Acidobacteriota* (Figure 5A).

At the order level (Figure 5B), *Burkholderiales* with members known to participate in nitrification, denitrification and also phosphorus metabolism, showed associations with *Chitinophagales, Bacteroidales, Blastocatellales, Anaerolineales, Ignavibacteriales* and *Pedosphaerales* that, in turn, include taxa that participate in carbon fermentation and degradation of carbon compounds like chitin, cellulose, mucin, hydrocarbons, organic matter (Belova et al., 2018; Herrmann et al., 2019; Campbell et al., 2014; Wang et al., 2022). Previous literature has established that in environments characterized by oxygen limitation or complete absence of oxygen, nitrogen-respiring bacteria may utilize fermentation products as electron donors, thereby facilitating both the transformation of organic matter and nitrogen removal (Van Den Berg et al., 2017). This closely integrates the functional processes involved in nitrogen cycle with the carbon cycle. Since the readily available carbon fermentation products may act as electron donors in the reactions for reduction of nitrates and sulphates in the stabilization pond as well as the vegetated wetland, the microbial taxa involved in these processes are likely to be associated with each other in microbiomes. While further research is needed into mechanisms enabling microbial associations in NHBP-ICW, similar patterns of co-occurrence have been observed for these bacterial functional groups in previous studies on ecosystems related to wetlands and sewage (Wang et al., 2021). Interestingly, many bacterial orders, from this study, were also predicted to be influenced by levels of PO_4_-P, and/or TDS, suggesting the possible role of their interactions in remediation of influent sewage (Figure 6). Moreover, major nodes in the network observed in this study (Figure 5) belonged to OTUs resembling *Saccharimonas* and *Desulfoprunum* known for obligate fermentative metabolism and ability to utilize fermentation products for reduction of sulphates (Junghare and Schink, 2015). Also, notable was *Nitrospira* (Figure 5), which can couple oxidation of fermentation products with nitrate reduction to remain active in low dissolved oxygen conditions (Koch et al., 2015). Additionally, several dominant bacterial taxa formed associations with unclassified bacterial OTUs (Figure 5) which points toward the unique and uncharacterized microbial diversity at NHBP-ICW and their putative interactions that are yet to be explored.

While this study provides predictions for functions and putative processes in microbiomes associated with samples from distinct components of NHBP-ICW that differed in water-quality parameters, various limitations and areas of future research need to be mentioned. Longitudinal sample collections with large number of samples that capture the seasonal dynamics and site-variability inherent in the NHBP-ICW system is needed. Though out of scope in this study, functional predictions for microbial associations in complex sewage-rich environments inferred from dedicated microbial databases can be further validated using techniques such as shotgun metagenomics, transcriptomics, quantitative PCRs, metabolomics, and/or enzyme assays for better understanding of the amelioration of sewage water in NHBP-ICW. Integration of multi-omics approaches, functional assays and molecular methods can help to address functional database limitations and the biases common in 16S rRNA gene-based metagenomic sequencing data (Ortiz-Estrada et al., 2018; Yi et al., 2020). Such targeted studies would provide a definitive assessment of actual metabolic activity for specific functional groups and their subsequent ecological interactions occurring in microbial communities within the wetland systems.

## Conclusion

Since constructed wetlands provide a practical substitute for the conventional wastewater treatment using various physical, chemical and biological processes, a thorough assessment of the microbial community structure, diversity and function within CWs becomes crucial to optimize CW performance and efficiency in reducing pollution. The findings of this study help to identify the potential microbial functions that enable amelioration of wastewater in the complex CW system. Further research into NHBP-ICW stabilization pond sludge is needed to validate the metabolic dexterity of microbes involved in carbon fermentation, sulphate respiration and methanogenesis that can facilitate transformation of copious organic matter and breakdown of complex pollutants in the influents under anoxic conditions. The rhizospheric sediments from vegetated wetland site of NHBP-ICW can be examined for bacterial groups involved in aerobic nitrate and nitrite respiration. Albeit predictive, an interesting and potentially useful result from this study, is the identification of distinct associations and underlying complex, metabolic cross-talk within and among diverse bacterial groups in samples from the NHBP-ICW. It is currently not clear if these diverse and complex microbial functional associations are indicative of the maturity of ICW ecosystem, established populations of perennial aquatic hydrophytes in the vegetated wetlands and/or consistent nature of influent sewage.

Although this study uses 16S rRNA gene metagenomic dataset with limited samples from contrasting components of NHBP-ICW, nonetheless, the predicted microbial associations can be validated by quantification of genes involved in various functional processes and/or use of transcriptomics and shotgun metagenomics with larger number of spatiotemporal samplings. Further work involving isolation and culture of the taxa associated with changes in sewage quality can facilitate the development of microbial consortia to enhance the removal efficiencies for pollutants in CWs. The functional profiles of the CW microbiomes, based on computational methods can aid identification of microbial activities in specific zones of the constructed wetland by inducing favorable conditions such as aeration, managing water hydrology or regulating organic loadings. Strategies addressing efficient management of sludge and sediments that minimize the release of greenhouse gases can be implemented to further enhance the functioning of similar CWs. Despite the limitations in the number of spatial and temporal samplings as well as reliance on 16S functional predictions and correlation networks, nevertheless, the findings from this study indicate the complexity of interactions between diverse microbial groups involved in metabolic pathways that rejuvenate water quality and sustain the NHBP-ICW. The outcomes of this study can be targeted to build a predictive framework for optimising sustainable, nature-based solutions while highlighting the application of metagenomic analysis to gain insights for microbe-mediated pollutant removal from STP effluents and wastewaters in constructed wetlands.

## Data Availability

The datasets presented in this study can be found in online repositories. The names of the repository/repositories and accession number(s) can be found in the article/Supplementary material.

## Supplementary material

Supplementary Figure 1 Relative abundance of bacterial genera known to be involved in removal of **(A)** nitrogen and phosphorus and **(B)** antibiotics, heavy metals and other emergent pollutants as described in literature.

Supplementary Figure 2 Heatmap showing Spearman correlation coefficient computed for bacterial **(A)** orders and **(B)** genera with different sewage water quality parameters. Only bacterial taxa with positive correlations with physicochemical parameter are shown here (denoted as: ^*^*p* < 0.05; ^**^*p* < 0.01).

Supplementary Figure 3 Comparison of sewage water quality parameters **(A–G)** between stabilization pond sludge and rhizospheric sediment samples from vegetated wetland unit in NHBP-ICW.

Supplementary Figure 4 Results of CCA for microbial community composition for stabilization pond sludge and rhizospheric sediment samples at **(A)** phylum and **(B)** genera levels (Top 10 taxa) with scores of PC1 for sewage water quality parameters represented as composite environmental gradient PC1.

Supplementary Table 1 Results of PCA analyses showing values of extracted principal components and loadings, eigenvalues, proportion of variance and cumulative proportion of variances explained by first six principal components for different physicochemical parameters.

Supplementary Table 2 Results of eigenvalues and proportion of variance explained by different physicochemical parameters.
