# Supplementary figures and images for: Functional diversity in bacterial communities of an integrated constructed wetland used for in situ bioremediation of sewage
Source: Front Microbiol. 2026 May 28;17:1803785. doi: 10.3389/fmicb.2026.1803785 (PMC13255553; doi:10.3389/fmicb.2026.1803785)

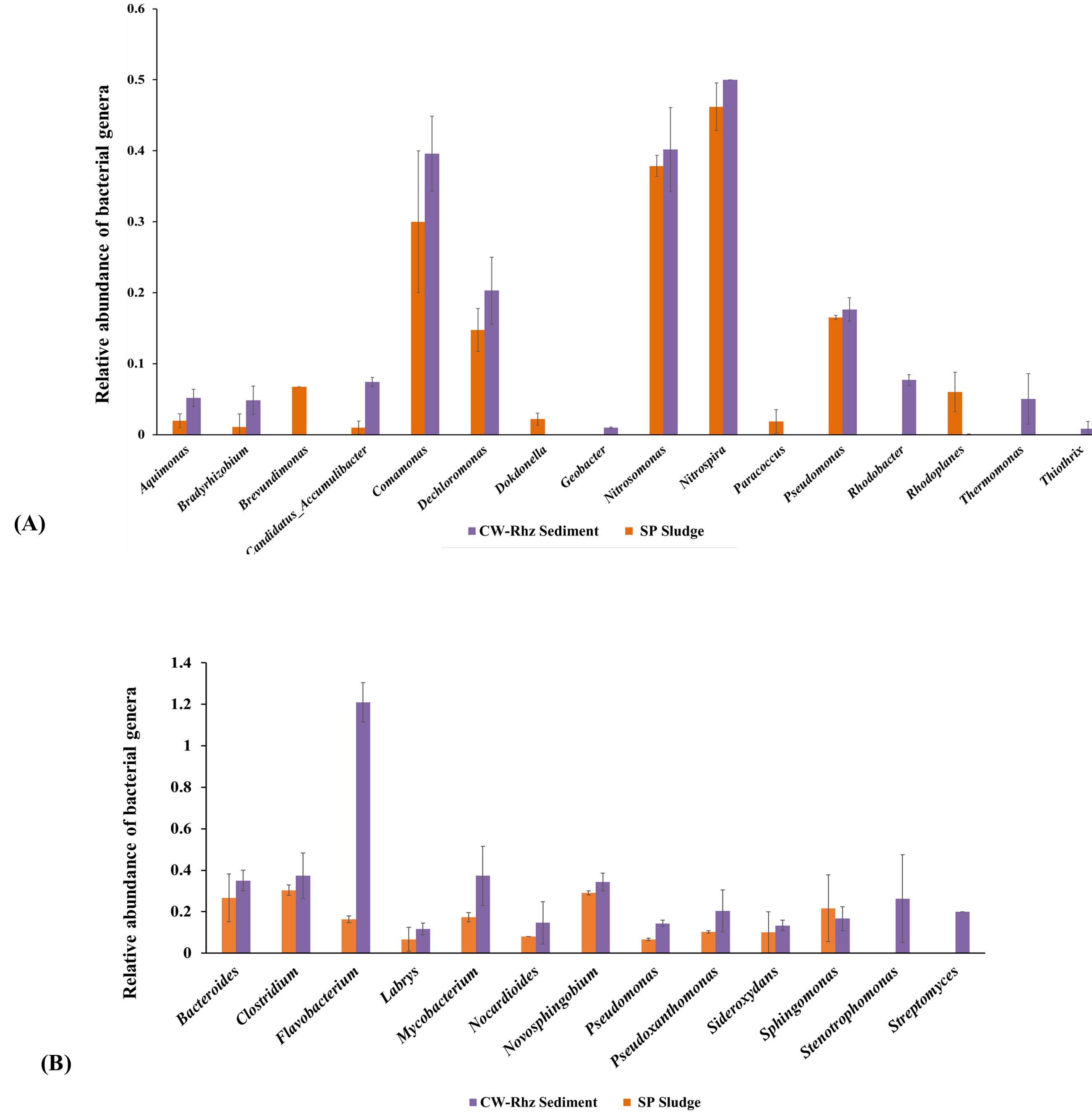

Supplement: Supplementary Figure 1 — Relative abundance of bacterial genera known to be involved in removal of (A) nitrogen and phosphorus and (B) antibiotics, heavy metals and other emergent pollutants as described in literature. [file Image_1.tif]

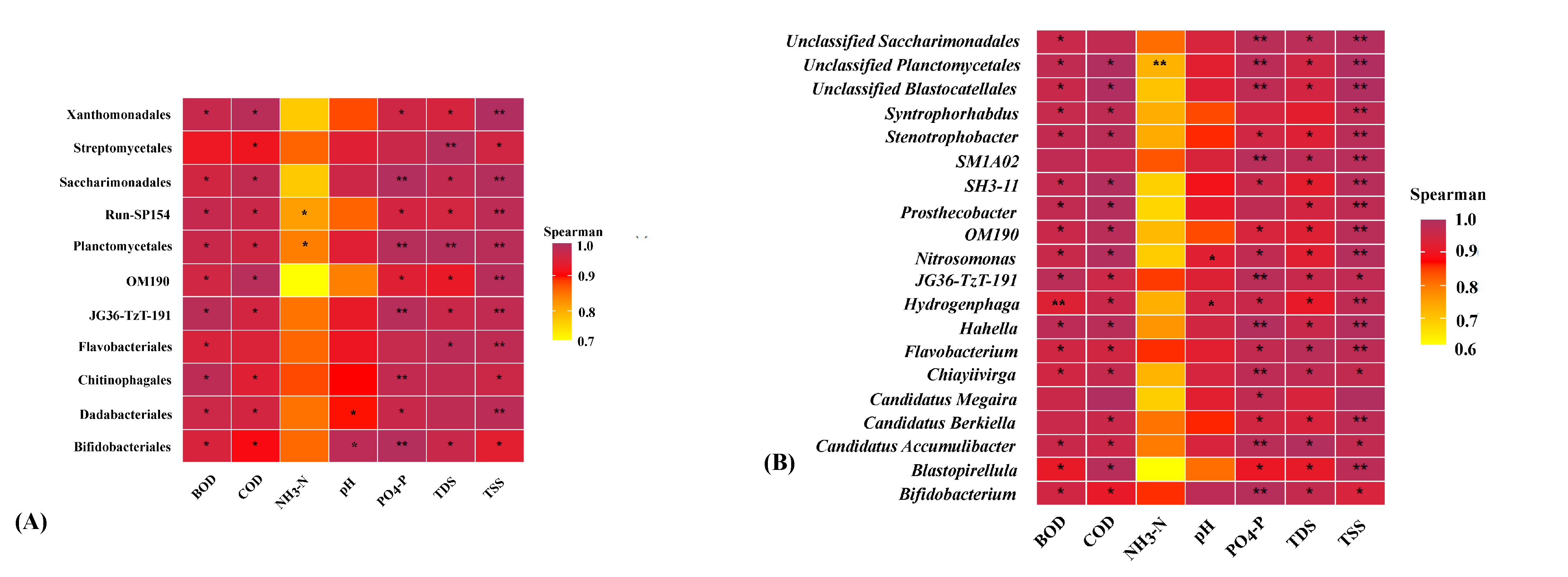

Supplement: Supplementary Figure 2 — Heatmap showing Spearman correlation coefficient computed for bacterial (A) orders and (B) genera with different sewage water quality parameters. Only bacterial taxa with positive correlations with physicochemical parameter are shown here (denoted as: *p < 0.05; **p < 0.01). [file Image_2.tif]

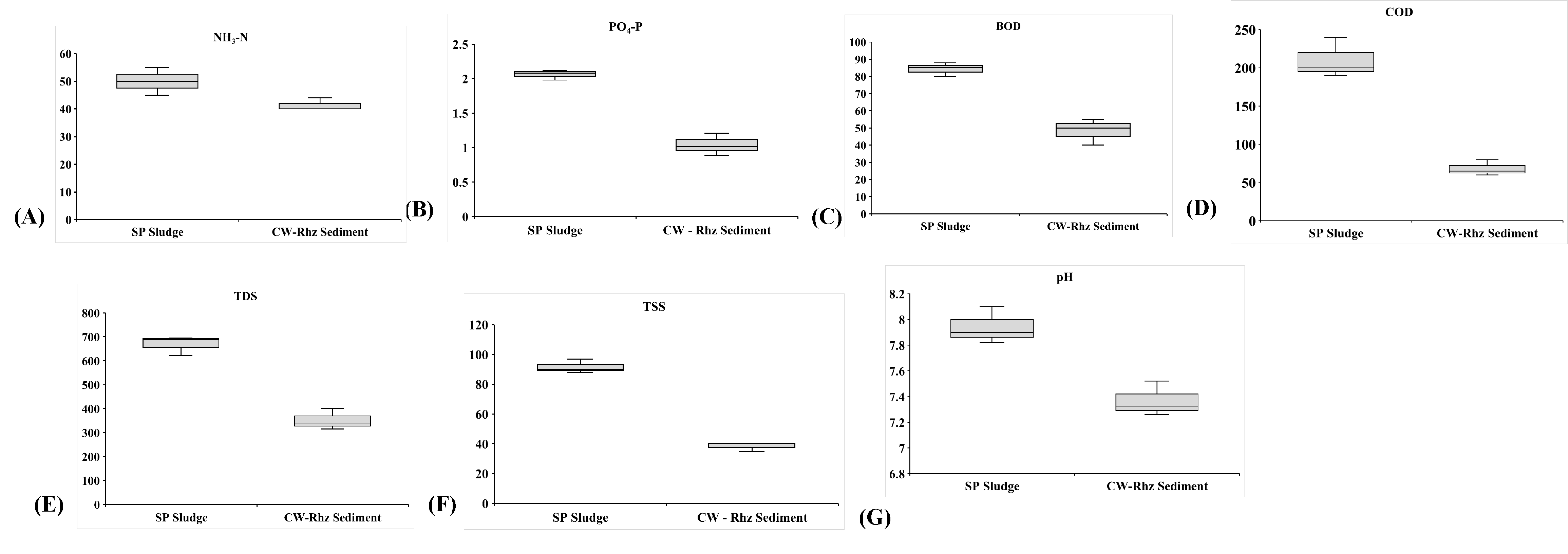

Supplement: Supplementary Figure 3 — Comparison of sewage water quality parameters (A–G) between stabilization pond sludge and rhizospheric sediment samples from vegetated wetland unit in NHBP-ICW. [file Image_3.tif]

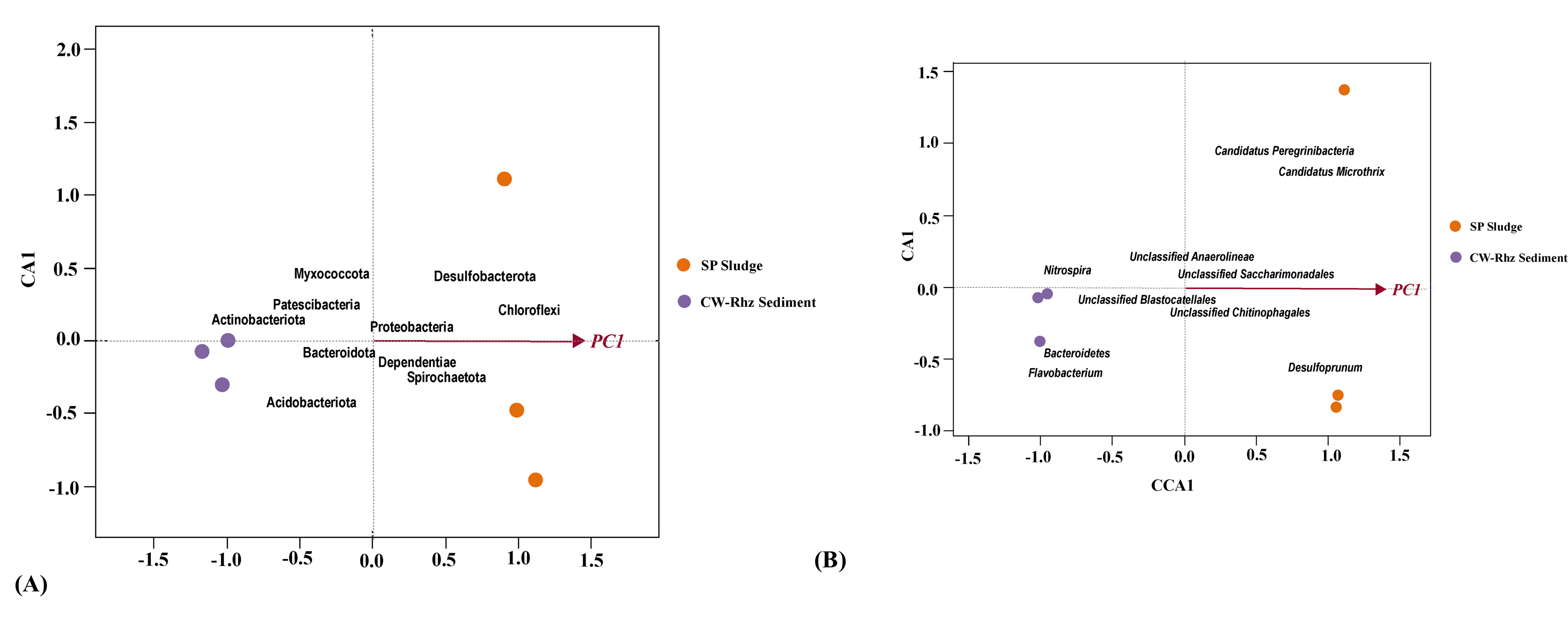

Supplement: Supplementary Figure 4 — Results of CCA for microbial community composition for stabilization pond sludge and rhizospheric sediment samples at (A) phylum and (B) genera levels (Top 10 taxa) with scores of PC1 for sewage water quality parameters represented as composite environmental gradient PC1. [file Image_4.tif]
