# Supplementary material for: Functional diversity in bacterial communities of an integrated constructed wetland used for in situ bioremediation of sewage
Source: Front Microbiol. 2026 May 28;17:1803785. doi: 10.3389/fmicb.2026.1803785 (PMC13255553; doi:10.3389/fmicb.2026.1803785)
Supplement: Supplementary Table 1 — Results of PCA analyses showing values of extracted principal components and loadings, eigenvalues, proportion of variance and cumulative proportion of variances explained by first six principal components for different physicochemical parameters. [file Table_1.docx]

Supplementary Table 1: Results of PCA analyses showing values of extracted principal components and loadings, eigenvalues, proportion of variance and cumulative proportion of variances explained by first six principal components for different physicochemical parameters

| **Env. factor** | **PC1** | **PC2** | **PC3** | **PC4** | **PC5** | **PC6** |
| --- | --- | --- | --- | --- | --- | --- |
| **BOD** | -0.3878 | 0.06685 | -0.1669 | 0.75612 | 0.23291 | -0.1895 |
| **NH_3_-N** | -0.33 | **-0.8653** | -0.1526 | 0.02086 | -0.3077 | -0.0739 |
| **PO_4_-P** | -0.3937 | 0.06545 | 0.20778 | 0.20011 | 0.17112 | 0.72795 |
| **pH** | -0.3757 | 0.01064 | 0.84916 | -0.1446 | -0.0523 | -0.302 |
| **COD** | -0.38 | **0.41275** | -0.2526 | -0.0185 | -0.2598 | -0.4615 |
| **TSS** | -0.3868 | **0.25916** | -0.2235 | -0.2852 | -0.5285 | 0.34283 |
| **TDS** | -0.388 | -0.0702 | -0.2662 | -0.534 | 0.68726 | -0.0839 |
| ***Eigenvalues*** | 2.5263 | 0.63249 | 0.36868 | 0.24229 | 0.15229 | 2.113e-17 |
| ***Proportion of variance*** | 0.9117 | 0.05715 | 0.01942 | 0.00839 | 0.00331 | 0.000 |
| ***Cumulative proportion*** | 0.9117 | 0.96888 | 0.98830 | 0.99669 | 1.00 | 1.00 |

| **Env. factor** | **NH_3_-N** | **COD** | **TSS** | **PO_4_-P** | **TDS** | **BOD** | **pH** |
| --- | --- | --- | --- | --- | --- | --- | --- |
| **Eigenvalues** | 0.858 | 0.315 | 0.217 | 0.159 | 0.155 | 0.155 | 0.141 |
| **Proportion of variance** | 0.429 | 0.157 | 0.108 | 0.080 | 0.078 | 0.077 | 0.071 |

Supplementary Table 2: Results of eigenvalues and proportion of variance explained by different physicochemical parameters
